# Supplementary material for: Mannose metabolism inhibition sensitizes acute myeloid leukaemia cells to therapy by driving ferroptotic cell death
Source: Nat Commun. 2023 Apr 14;14:2132. doi: 10.1038/s41467-023-37652-0 (PMC10104861; doi:10.1038/s41467-023-37652-0)
Supplement: Supplementary file 1 — Supplementary Information [file 41467_2023_37652_MOESM1_ESM.pdf]

**Mannose metabolism inhibition sensitizes acute myeloid leukemia cells to therapy by driving ferroptotic cell death**

Woodley et al.

Supplementary Information File containing:

Supplementary Figures 1-10

FACS gating strategies

Supplementary data 1 and 2 (in separate files)

Supplementary Table 1

Full Western Blot membranes (uncropped and unprocessed scans of the western blots shown in the supplementary figures)

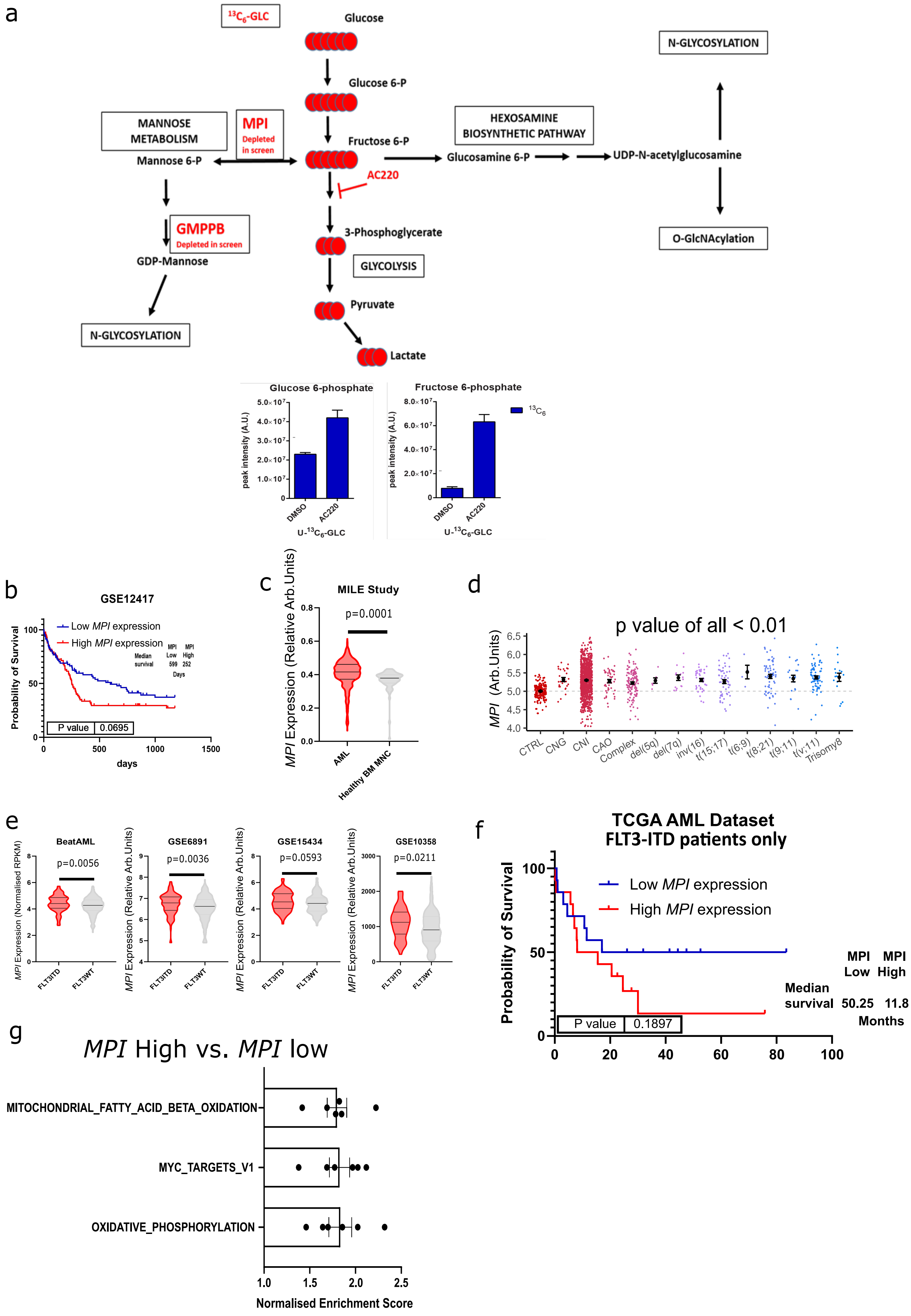

a
$^{13}C_6$-GLC
Glucose
Glucose 6-P
Fructose 6-P
MANNOSE METABOLISM
MPI Depleted in screen
Mannose 6-P
GMPPB Depleted in screen
GDP-Mannose
N-GLYCOSYLATION
AC220
3-Phosphoglycerate
GLYCOLYSIS
Pyruvate
Lactate
HEXOSAMINE BIOSYNTHETIC PATHWAY
Glucosamine 6-P
UDP-N-acetylglucosamine
N-GLYCOSYLATION
O-GlcNAcylation
Glucose 6-phosphate
Fructose 6-phosphate
peak intensity (A.U.)
DMSO
AC220
U-$^{13}C_6$-GLC
$^{13}C_6$
b
GSE12417
Low MPI expression
High MPI expression
Median survival
MPI Low 599
MPI High 252
Days
Probability of Survival
P value 0.0695
days
c
MILE Study
p=0.0001
MPI Expression (Relative Arb.Units)
AML
Healthy BM MNC
d
p value of all < 0.01
MPI (Arb.Units)
CTRL CNG CNI CAO Complex del(5q) del(7q) inv(16) t(15;17) t(6;9) t(8;21) t(9;11) t(v;11) Trisomy8
e
BeatAML p=0.0056
GSE6891 p=0.0036
GSE15434 p=0.0593
GSE10358 p=0.0211
MPI Expression (Normalised RPKM)
MPI Expression (Relative Arb.Units)
FLT3ITD
FLT3WT
f
TCGA AML Dataset
FLT3-ITD patients only
Low MPI expression
High MPI expression
Probability of Survival
P value 0.1897
MPI Low
MPI High
Median survival 50.25 11.8
Months
g
MPI High vs. MPI low
MITOCHONDRIAL_FATTY_ACID_BETA_OXIDATION
MYC_TARGETS_V1
OXIDATIVE_PHOSPHORYLATION
Normalised Enrichment Score

**Supplementary Figure 1**
**MPI is more highly expressed in AML cells compared to normal bone marrow**

**a** – Schematic of glycolysis and mannose metabolism with targets of AC220 and location of MPI and the downstream enzyme GMPPB (also depleted in reference 9) highlighted (top). Levels of glycolysis intermediates in MV411 cells treated with 1nM AC220 or vehicle grown in media containing uniformly-labelled $^{13}$Carbon(U-$^{13}C_6$)-glucose(GLC) measured by LC-MS analysis (bottom – data produced in reference 9); **b** - Kaplan-Meier curves comparing survival of patient from the GSE12417 dataset separated by the top 50% and bottom 50% of *MPI* expression. Log-rank (Mantel-Cox) test; **c** – Violin plots of *MPI* expression (relative) in AML samples compared to healthy bone marrow mononuclear cell samples from MILE (GSE13204) study. Unpaired t-test, two sided, N=73 for healthy BM MNC, N=542 for AML; **d** – Levels of *MPI* RNA in various AML subtypes when compared to control (CTRL) from GSE147515. N=198 for control, N=47 for CAO (cytogenetically abnormal, not otherwise specified), N=27 for CNG (cytogenetically normal, good prognosis), N=1043 for CNI (cytogenetically normal, intermediate prognosis), N=130 for complex, N=7 for del(5q), N=15 for del(7q), N=40 for inv(16), N=54 for t(15;17), N=4 for t(6;9), N=62 for t(8;21), N=19 for t(9;11), N=70 for t(v;11) and N=16 for Trisomy8, Mann-Whitney U test compared to control. **e** - Violin plots of *MPI* expression (normalised or relative) in FLT3-ITD AML samples compared to FLT3 WT AML samples from Beat AML dataset (left), GSE6891 dataset (middle left), GSE15434 dataset (middle right) and GSE10358 dataset (right). Unpaired t-test, N=105 Flt3-ITD, N=346 for Flt3 WT for BeatAML, N=196 Flt3-ITD, N=291 for Flt3 WT for GSE6891, N=90 Flt3-ITD, N=161 for Flt3 WT for GSE15434, N=56 Flt3-ITD, N=143 for Flt3 WT for GSE10358; **F** – Kaplan-Meier curves comparing survival of patient from the FLT3-ITD patients in the TCGA dataset separated by the top 50% and bottom 50% of *MPI* expression. Log-rank (Mantel-Cox) test N=14 for both low and high MPI; **G** - 3 significantly enriched gene signatures in *MPI* high expressing samples from 6 AML datasets (GSE6891, E-TABM1029, GSE12417, GSE15434, TCGA, and GSE13159) combined. Violin plots show median and quartiles.

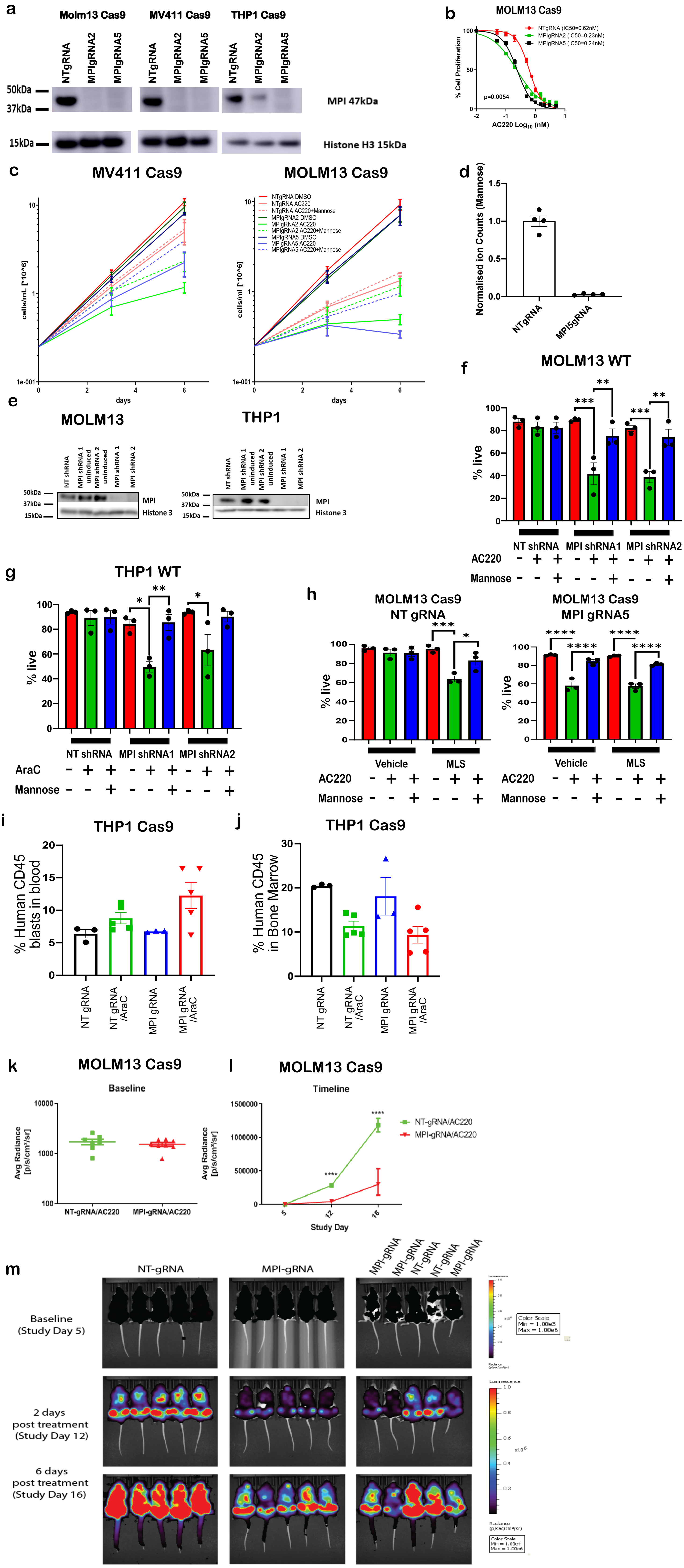
a
Molm13 Cas9
MV411 Cas9
THP1 Cas9
NTgRNA
MPIgRNA2
MPIgRNA5
50kDa
37kDa
15kDa
MPI 47kDa
Histone H3 15kDa
b
MOLM13 Cas9
NTgRNA (IC50=0.62nM)
MPIgRNA2 (IC50=0.23nM)
MPIgRNA5 (IC50=0.24nM)
% Cell Proliferation
p=0.0054
AC220 Log10 (nM)
c
MV411 Cas9
MOLM13 Cas9
NTgRNA DMSO
NTgRNA AC220
NTgRNA AC220+Mannose
MPIgRNA2 DMSO
MPIgRNA2 AC220
MPIgRNA2 AC220+Mannose
MPIgRNA5 DMSO
MPIgRNA5 AC220
MPIgRNA5 AC220+Mannose
cells/mL [*10^6]
cells/ml [*10^6]
days
d
Normalised ion Counts (Mannose)
NTgRNA
MPI5gRNA
e
MOLM13
THP1
NT shRNA
MPI shRNA 1 uninduced
MPI shRNA 2 uninduced
MPI shRNA 1
MPI shRNA 2
MPI
Histone 3
f
MOLM13 WT
% live
NT shRNA
MPI shRNA1
MPI shRNA2
AC220
Mannose
g
THP1 WT
% live
NT shRNA
MPI shRNA1
MPI shRNA2
AraC
Mannose
h
MOLM13 Cas9
NT gRNA
MOLM13 Cas9
MPI gRNA5
% live
Vehicle
MLS
AC220
Mannose
i
THP1 Cas9
% Human CD45 blasts in blood
NT gRNA
NT gRNA /AraC
MPI gRNA
MPI gRNA /AraC
j
THP1 Cas9
% Human CD45 in Bone Marrow
NT gRNA
NT gRNA /AraC
MPI gRNA
MPI gRNA /AraC
k
MOLM13 Cas9
Baseline
Avg Radiance [p/s/cm²/sr]
NT-gRNA/AC220
MPI-gRNA/AC220
l
MOLM13 Cas9
Timeline
Avg Radiance [p/s/cm²/sr]
NT-gRNA/AC220
MPI-gRNA/AC220
Study Day
m
NT-gRNA
MPI-gRNA
MPI-gRNA
MPI-gRNA
NT-gRNA
NT-gRNA
MPI-gRNA
Baseline (Study Day 5)
2 days post treatment (Study Day 12)
6 days post treatment (Study Day 16)
Luminescence
Radiance (p/sec/cm²/sr)
Color Scale
Min = 1.00e3
Max = 1.00e6
Color Scale
Min = 1.00e4
Max = 1.00e6

**Supplementary figure 2**
**MPI CRISPR KO, shRNA KD and chemical inhibition show a consistent phenotype**

**a** – Western blot for MPI in MOLM13, MV411 and THP1 cells transduced with NTgRNA, MPIgRNA2 and MPIgRNA5 showing MPI with Histone H3; **b** – IC50 dose curves for AC220 dosing of MOLM13 NTgRNA, MPIgRNA2 or MPIgRNA5. RM 1-way ANOVA for treatment effect, N=3; **c** – Cell proliferation of MV411 and MOLM13 NTgRNA, MPIgRNA2 or MPIgRNA5 cells treated with vehicle, AC220 or AC220 and mannose, N=3; **d** – Normalised ion counts showing intracellular mannose levels of NTgRNA and MPIgRNA5 MOLM13 cells from metabolomics, N=4; **e** – Western blot for MPI in MOLM13 (left) and THP1 (right) cells transduced with NTshRNA, MPIshRNA1 uninduced, MPIshRNA2 uninduced, MPIshRNA1 induced, MPIshRNA2 induced showing MPI with Histone H3. **f** - Percentage of live cells of WT MOLM13 cells transduced with either NTshRNA, MPIshRNA1 or MPIshRNA2 treated with vehicle, AC220 or mannose as indicated for 6 days, N=3, 1-way Anova with Tukey's correction; **g** - Percentage of live NTshRNA, MPIshRNA1 and MPIshRNA2 THP1 cells treated with vehicle, mannose, AraC or AraC and mannose for 3 days, N=4 for MV411, N=3 for MOLM13, 1-way Anova with Tukey's correction; **h** - Percentage of live cells of MOLM13 NTgRNA (left) and MPIgRNA5 (right) cells treated with vehicle, MLS0315771, AC220, mannose or in combinations as indicated. Treated for 6 days, N=3, 1-way Anova with Tukey's correction; **i** – Percentage of human CD45+ (THP1) cells in peripheral blood of NBSGW mice prior to the start of Cytarabine treatment, N=3 for control groups, N=5 for treated groups; **j** – Percentage of human CD45+ (THP1) cells in bone marrow of NBSGW mice at terminal timepoint, N=3 control groups, N=5 treated groups; **k** – Engraftment of NTgRNA and MPIgRNA5 MOLM13 cells in NSG mice measured by bioluminescence on day 5 post-transplant, N=8; **l** – Bioluminescence of NTgRNA and MPIgRNA5 MOLM13 cells engrafted into NSG mice over time after 5 days of treatment with AC220. Unpaired t-test, N=8; **m** – Visual representation of luminescence of NSG mice engrafted with NT gRNA or MPI gRNA5 MOLM13 cells at baseline and after treatment with AC220 (5mg/kg) over time. For all panels, ns = not significant, *=p<0.05, **=p<0.01, ***=p<0.005, ****=p<0.001, data presented as mean +/- SEM.

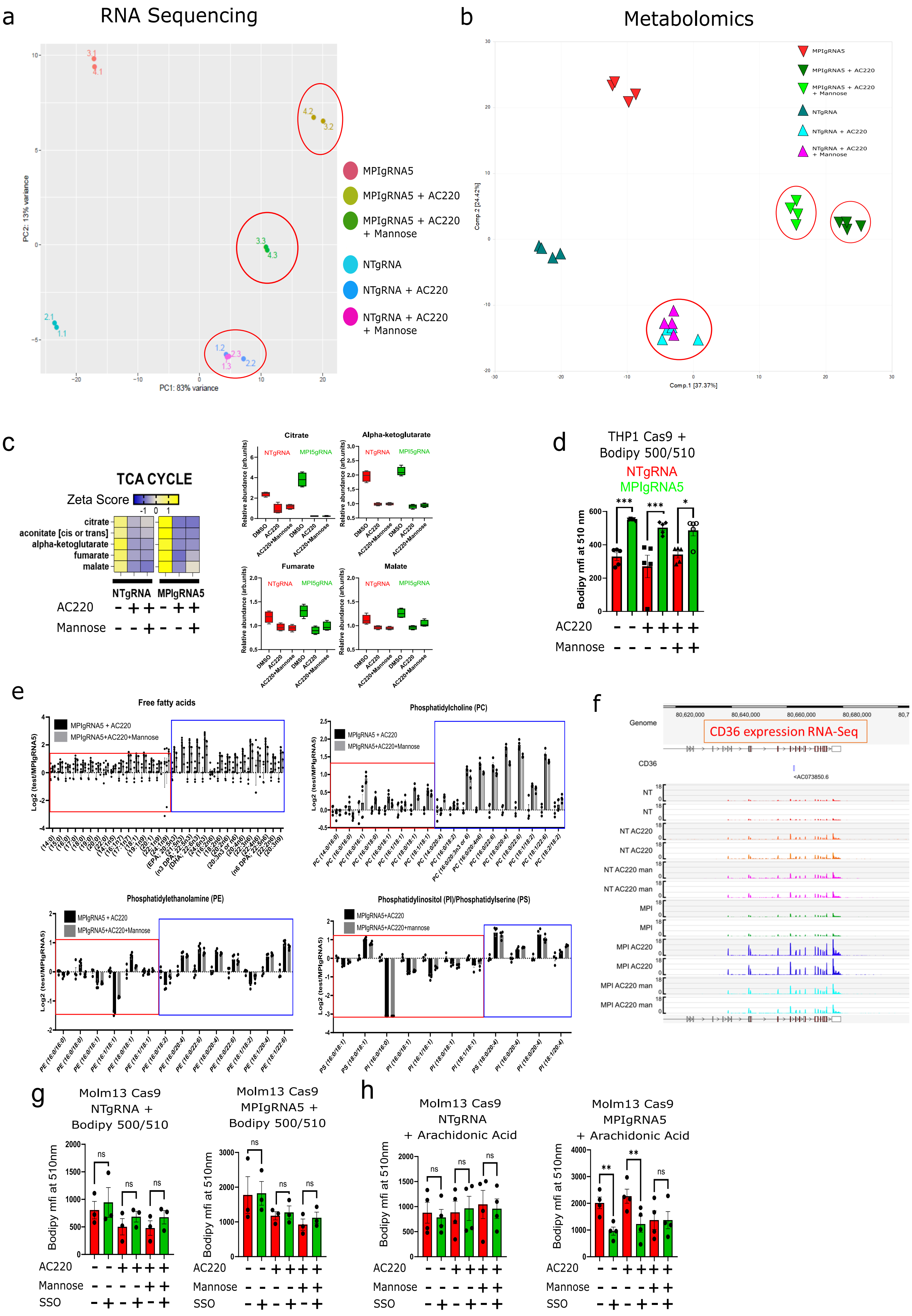

a
RNA Sequencing
PC1: 83% variance
PC2: 13% variance
MPIgRNA5
MPIgRNA5 + AC220
MPIgRNA5 + AC220 + Mannose
NTgRNA
NTgRNA + AC220
NTgRNA + AC220 + Mannose
b
Metabolomics
Comp.1 [37.37%]
Comp.2 [24.42%]
MPIgRNA5
MPIgRNA5 + AC220
MPIgRNA5 + AC220 + Mannose
NTgRNA
NTgRNA + AC220
NTgRNA + AC220 + Mannose
c
TCA CYCLE
Zeta Score
citrate
aconitate [cis or trans]
alpha-ketoglutarate
fumarate
malate
NTgRNA
MPIgRNA5
AC220
Mannose
Citrate
Alpha-ketoglutarate
Fumarate
Malate
Relative abundance (arb.units)
NTgRNA
MPI5gRNA
DMSO
AC220
AC220+Mannose
d
THP1 Cas9 +
Bodipy 500/510
NTgRNA
MPIgRNA5
Bodipy mfi at 510 nm
AC220
Mannose
e
Free fatty acids
Phosphatidylcholine (PC)
Phosphatidylethanolamine (PE)
Phosphatidylinositol (PI)/Phosphatidylserine (PS)
MPIgRNA5 + AC220
MPIgRNA5+AC220+Mannose
Log2 (test/MPIgRNA5)
f
Genome
CD36 expression RNA-Seq
CD36
NT
NT AC220
NT AC220 man
MPI
MPI AC220
MPI AC220 man
g
Molm13 Cas9
NTgRNA +
Bodipy 500/510
Molm13 Cas9
MPIgRNA5 +
Bodipy 500/510
Bodipy mfi at 510nm
AC220
Mannose
SSO
h
Molm13 Cas9
NTgRNA
+ Arachidonic Acid
Molm13 Cas9
MPIgRNA5
+ Arachidonic Acid
Bodipy mfi at 510nm
AC220
Mannose
SSO

**Supplementary figure 3**
**Increased FA uptake in MPI KO is not exclusively caused by CD36**

**a** – PCA of RNA sequencing data from NTgRNA and MPIgRNA5 MOLM13 cells treated with vehicle, AC220 or mannose as indicated for 24 hours. Red circles indicate NT gRNA with AC220 and AC220 and mannose, MPIgRNA5 with AC220 and AC220 and mannose; **b** – PCA of metabolomics data from NTgRNA and MPIgRNA5 MOLM13 cells treated with vehicle, AC220 or mannose as indicated for 48 hours. Circles indicate NTgRNA with AC220 and AC220 and mannose, MPIgRNA5 with AC220 and AC220 and mannose; **c** – Comparison of TCA cycle metabolites from metabolic profiling of MOLM13 NTgRNA and MPIgRNA5 cells treated with vehicle, AC220 or mannose as indicated for 24 hours with heat map and graphs of metabolites, N=4; **d** - Uptake of the fluorescent C1-Bodipy 500/510-C12 by NTgRNA or MPIgRNA5 THP1 cells treated with vehicle, AC220 or AC220 and mannose for 24 hours, with a representative flow cytometry plot (right). N=5, 1-way Anova with Tukey's correction; **e** – Levels of free fatty acid (FA) (top left), phosphatidylcholine (top right), phosphatidylethanolamine (bottom left) and phosphatidylinositol/phosphatidylserine (bottom right) from global metabolomics of MPIgRNA5 cells treated with AC220 or AC220 and mannose for 48 hours. Saturated or monounsaturated FAs are in the red boxes, polyunsaturated FAs are in the blue boxes, N=4; **f** – Expression of CD36 from RNA sequencing in NTgRNA and MPIgRNA5 MOLM13 cells treated with vehicle, AC220 or mannose as indicated for 48 hours; **g** - Uptake of the fluorescent C1-Bodipy 500/510-C12 by NTgRNA (left panel) or MPIgRNA5 (right panel) MOLM13 cells treated with SSO or vehicle for 24 hours then vehicle, AC220, mannose or in combinations as indicated with C1-Bodipy 500/510-C12 for a further 24 hours. N=3, 1-way Anova with Tukey's correction; **h** - Uptake of arachidonic acid by NTgRNA (left panel) or MPIgRNA5 (right panel) MOLM13 cells treated with SSO or vehicle for 24 hours then vehicle, AC220 or mannose as indicated with arachidonic acid for a further 24 hours. N=4, 1-way Anova with Tukey's correction. For all panels, ns = not significant, *=p<0.05, **=p<0.01, ***=p<0.005, ****=p<0.001, data presented as mean +/- SEM, box plots presented as median with upper and lower quartiles as bounds of box and whiskers as max and min of distribution.

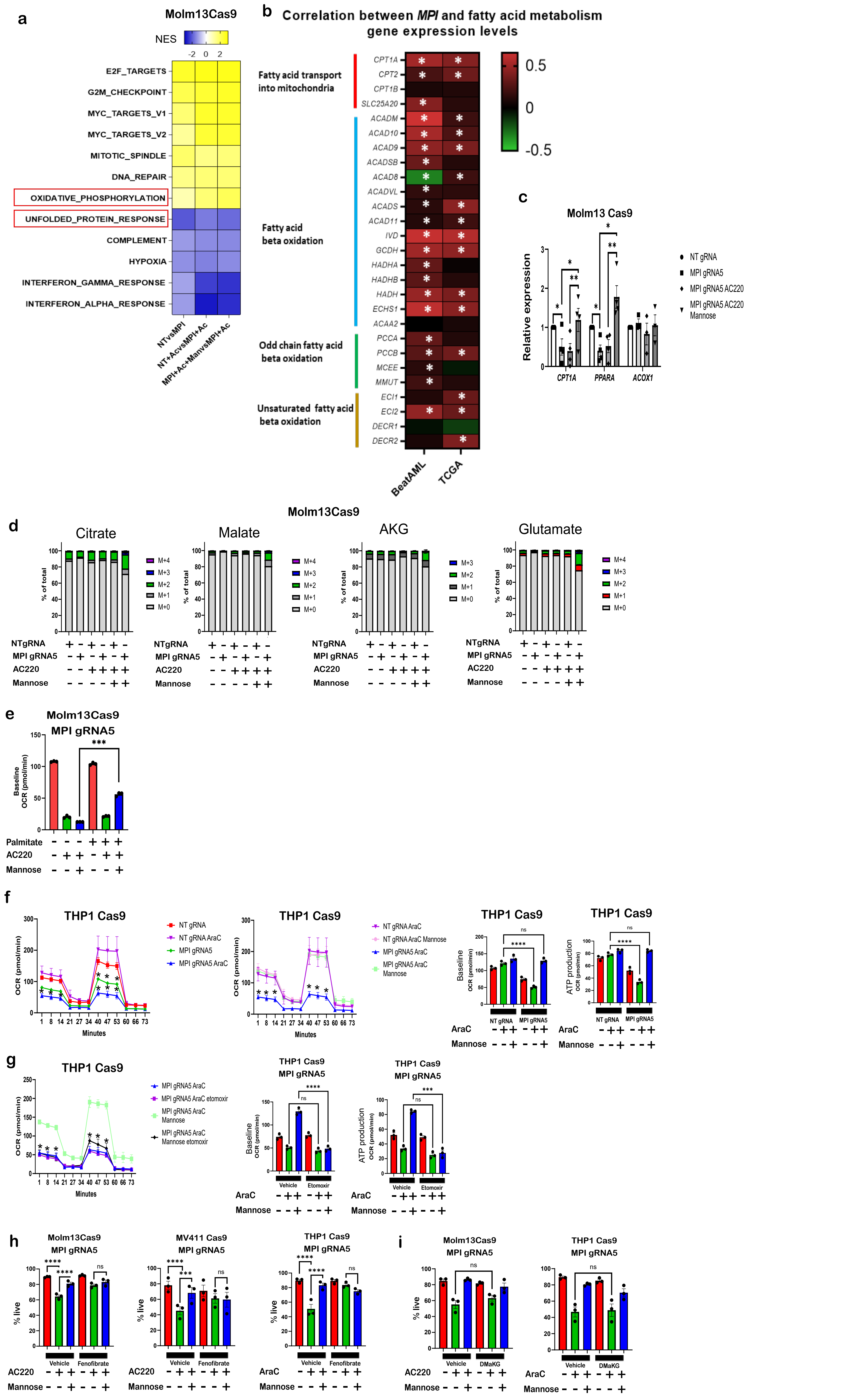

a
Molm13Cas9
NES
-2 0 2
E2F_TARGETS
G2M_CHECKPOINT
MYC_TARGETS_V1
MYC_TARGETS_V2
MITOTIC_SPINDLE
DNA_REPAIR
OXIDATIVE_PHOSPHORYLATION
UNFOLDED_PROTEIN_RESPONSE
COMPLEMENT
HYPOXIA
INTERFERON_GAMMA_RESPONSE
INTERFERON_ALPHA_RESPONSE
NTvsMPI
NT+AcvsMPI+Ac
MPI+AcvsMPI+Man+Ac
b
Correlation between MPI and fatty acid metabolism gene expression levels
Fatty acid transport into mitochondria
Fatty acid beta oxidation
Odd chain fatty acid beta oxidation
Unsaturated fatty acid beta oxidation
CPT1A
CPT2
CPT1B
SLC25A20
ACADM
ACAD10
ACAD9
ACADSB
ACAD8
ACADVL
ACADS
ACAD11
IVD
GCDH
HADHA
HADHB
HADH
ECHS1
ACAA2
PCCA
PCCB
MCEE
MMUT
ECI1
ECI2
DECR1
DECR2
BeatAML
TCGA
0.5
0
-0.5
c
Molm13 Cas9
Relative expression
CPT1A
PPARA
ACOX1
NT gRNA
MPI gRNA5
MPI gRNA5 AC220
MPI gRNA5 AC220 Mannose
d
Molm13Cas9
Citrate
Malate
AKG
Glutamate
% of total
M+4
M+3
M+2
M+1
M+0
NTgRNA
MPI gRNA5
AC220
Mannose
e
Molm13Cas9
MPI gRNA5
Baseline OCR (pmol/min)
Palmitate
AC220
Mannose
f
THP1 Cas9
OCR (pmol/min)
Minutes
NT gRNA
NT gRNA AraC
MPI gRNA5
MPI gRNA5 AraC
NT gRNA AraC Mannose
MPI gRNA5 AraC Mannose
Baseline OCR (pmol/min)
ATP production OCR (pmol/min)
NT gRNA
MPI gRNA5
AraC
Mannose
g
THP1 Cas9
MPI gRNA5 AraC
MPI gRNA5 AraC etomoxir
MPI gRNA5 AraC Mannose
MPI gRNA5 AraC Mannose etomoxir
THP1 Cas9
MPI gRNA5
Vehicle
Etomoxir
h
Molm13Cas9
MPI gRNA5
MV411 Cas9
MPI gRNA5
THP1 Cas9
MPI gRNA5
% live
Vehicle
Fenofibrate
AC220
AraC
Mannose
i
Molm13Cas9
MPI gRNA5
THP1 Cas9
MPI gRNA5
% live
Vehicle
DMaKG
AC220
AraC
Mannose

**Supplementary figure 4**
**MPI KO causes a reduction in fatty acid oxidation in AML cell lines**

**a** – Heatmap showing NES for most significantly up or downregulated hallmark genesets from RNA sequencing NTgRNA or MPIgRNA5 treated with AC220 or mannose as indicated; **b** – Heatmap showing correlation between *MPI* and FA metabolism genes from BeatAML and TCGA databases. Spearman's Rank correlation; **c** – Expression of *CPT1A*, *PPARA* and *ACOX1*, normalised to *ACTB* expression, from RT-qPCR, in NTgRNA and MPIgRNA5 MOLM13 cells treated with vehicle, AC220 or mannose as indicated for 72 hours. *ACOX1* is a control gene involved with peroxisomal lipid oxidation. N=4 for *CPT1a* and *PPARA*, N=3 for *ACOX1*, 1-way Anova with Tukey's correction; **d** - Percentage of TCA cycle and associated metabolites labelled with $^{13}C$ from 24 hour $^{13}C_{16}$-palmitate treatment with AC220 and mannose in MPI KO and NT MOLM13 cells. N=5; **e** - Baseline OCR of MPIgRNA5 cells cultured overnight in substrate limited media without FBS and glutamine treated with palmitate, vehicle, AC220, mannose as indicated from SEAHORSE MitoStress test. N=3, 1-way Anova with Tukey's correction; **f** - SEAHORSE MitoStress tests showing OCR comparing NTgRNA and MPIgRNA5 THP1 cells treated with vehicle, AraC or mannose as indicated after 72 hours, N=3, 2-way Anova with Sidak's correction (left panels). Baseline OCR and ATP production of NTgRNA and MPIgRNA5 THP1 cells treated with vehicle, AraC or mannose as indicated after 72 hours, N=2, 1-way Anova with Tukey's correction (right panels); **g** - SEAHORSE MitoStress tests showing OCR comparing MPIgRNA5 THP1 cells treated with etomoxir, AraC and mannose after 72 hours as indicated. N=2, 2-way Anova with Sidak's correction (left panel). Baseline OCR and ATP production of MPIgRNA5 THP1 cells treated with vehicle, etomoxir, AraC, mannose after 72 hours as indicated. N=2, 1-way Anova with Tukey's correction (right panels); **h** - Percentage of live MPIgRNA5 MOLM13, MV411 and THP1 cells treated with vehicle, fenofibrate, AC220 or mannose as indicated after 72 hours. N=3, 1 way Anova with Tukey's; **i** – Percentage of live MPIgRNA5 MOLM13 cells and THP1 treated with vehicle, DMaKG, AC220, mannose or combinations as indicated 72 hours after treatment. N=3, 1 way Anova with Tukey's correction. For all panels, ns = not significant, *=p<0.05, **=p<0.01, ***=p<0.005, ****=p<0.001, data presented as mean +/- SEM.

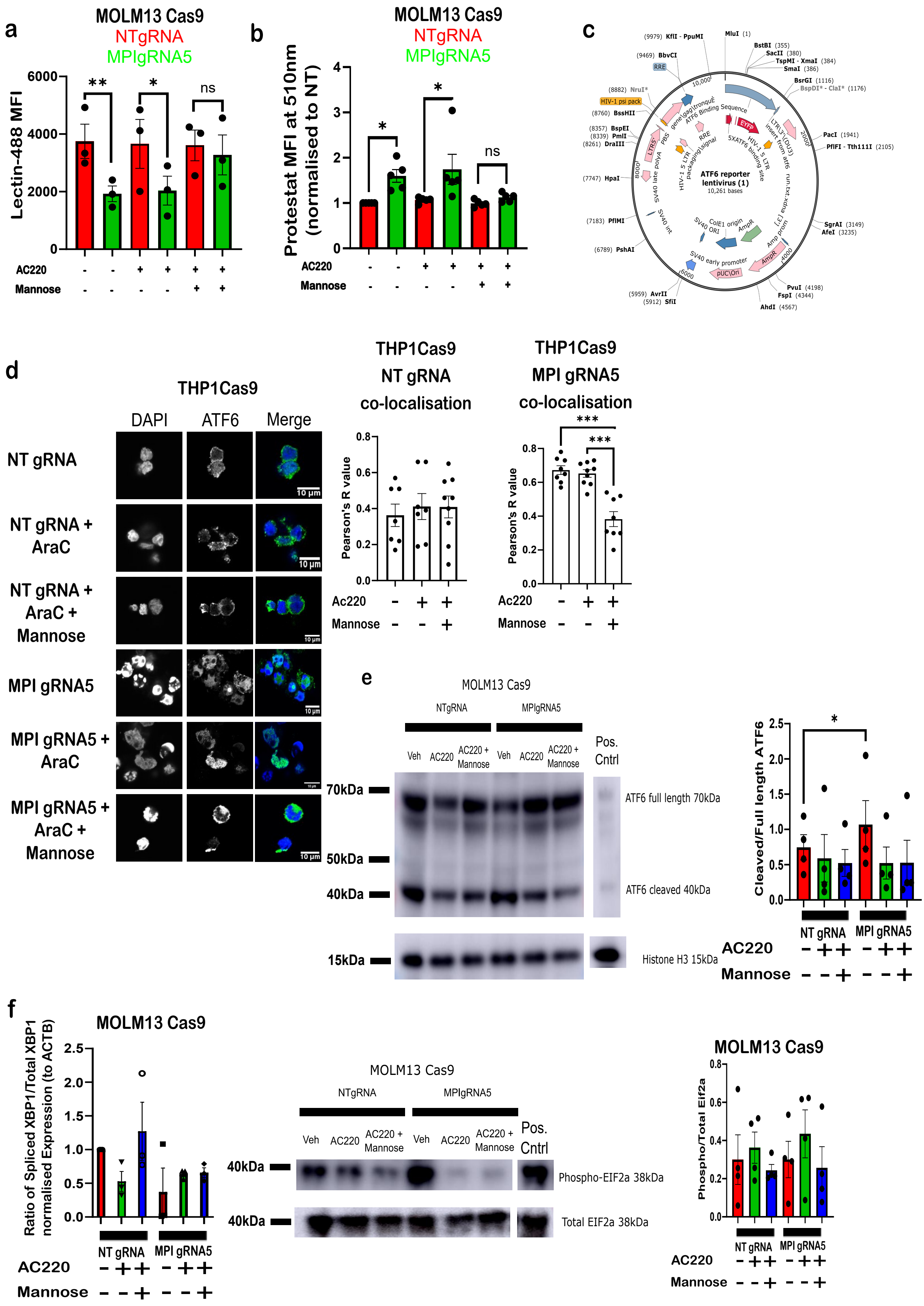
a
MOLM13 Cas9
NTgRNA
MPIgRNA5
Lectin-488 MFI
AC220
Mannose
b
Protestat MFI at 510nm (normalised to NT)
c
ATF6 reporter lentivirus (1)
10,261 bases
d
THP1Cas9
DAPI
ATF6
Merge
NT gRNA
NT gRNA + AraC
NT gRNA + AraC + Mannose
MPI gRNA5
MPI gRNA5 + AraC
MPI gRNA5 + AraC + Mannose
THP1Cas9 NT gRNA co-localisation
THP1Cas9 MPI gRNA5 co-localisation
Pearson's R value
Ac220
Mannose
e
MOLM13 Cas9
NTgRNA
MPIgRNA5
Veh
AC220
AC220 + Mannose
Pos. Cntrl
70kDa
50kDa
40kDa
15kDa
ATF6 full length 70kDa
ATF6 cleaved 40kDa
Histone H3 15kDa
Cleaved/Full length ATF6
NT gRNA
MPI gRNA5
AC220
Mannose
f
MOLM13 Cas9
Ratio of Spliced XBP1/Total XBP1 normalised Expression (to ACTB)
Phospho-EIF2a 38kDa
Total EIF2a 38kDa
Phospho/Total Eif2a

**Supplementary figure 5**
**ATF6 arm of the UPR is increased in MPI KO AML cells**

**a** – Lectin-488 MFI from flow cytometry of NTgRNA or MPIgRNA5 MOLM13 cells treated with vehicle, AC220 or AC220 and Mannose for 72 hours. N=3, 1-way Anova with Tukey's correction; **b –** Proteostat MFI from flow cytometry of NTgRNA or MPIgRNA5 MOLM13 cells treated with vehicle, AC220 or AC220 and Mannose for 72 hours. N=3, 1-way Anova with Tukey's correction; **c** – Plasmid map of the YFP-linked ATF6 reporter; **d** – Confocal microscopy images of NTgRNA and MPIgRNA5 THP1 cells stained for DAPI (blue, 1st column) and ATF6 (green, 2nd column) with a merged image (3rd column), treated with vehicle, AraC, mannose or in combinations as indicated after 72 hours (left). Colocalisation analysis from immunofluorescence images of NTgRNA (left) and MPIgRNA5 (right) THP1 cells treated with vehicle, AraC, mannose or in combinations as indicated. Analysis performed with Coloc2 plugin in ImageJ, ordinary 1-way Anova with Tukey's correction (right), experiment performed 3 times independently, N=7 for NTgRNA, N=9 for MPIgRNA5; **e** - Western blot of protein samples from NTgRNA or MPIgRNA5 MOLM13 cells treated with vehicle, AC220 or AC220 and mannose as indicated at 72 hours of treatment. Primary antibodies ATF6 and total Histone H3 as a loading control (Left). Densitometry of cleaved ATF6 divided full length ATF6 (right), N=4, 1-way Anova with Tukey's correction; **f -** Relative expression of spliced *XBP1* and total *XBP1* normalised to *ACTB* expression, from RT-qPCR, in NT gRNA and MPI gRNA5 MOLM13 cells treated with vehicle, AC220 or mannose in combinations as indicated for 72 hours (left panel). Western blot of protein samples from NT gRNA or MPI gRNA5 MOLM13 cells treated with vehicle, AC220 or AC220 and mannose as indicated at 72 hours of treatment. Primary antibodies EIF2α and phospho-EIF2α (middle panel) with densitometry analysis of phospho EIF2α divided by total EIF2α (right panel). N=3. For all panels, ns = not significant, *=p<0.05, **=p<0.01, ***=p<0.005, ****=p<0.001. All data presented as mean values +/- SEM.

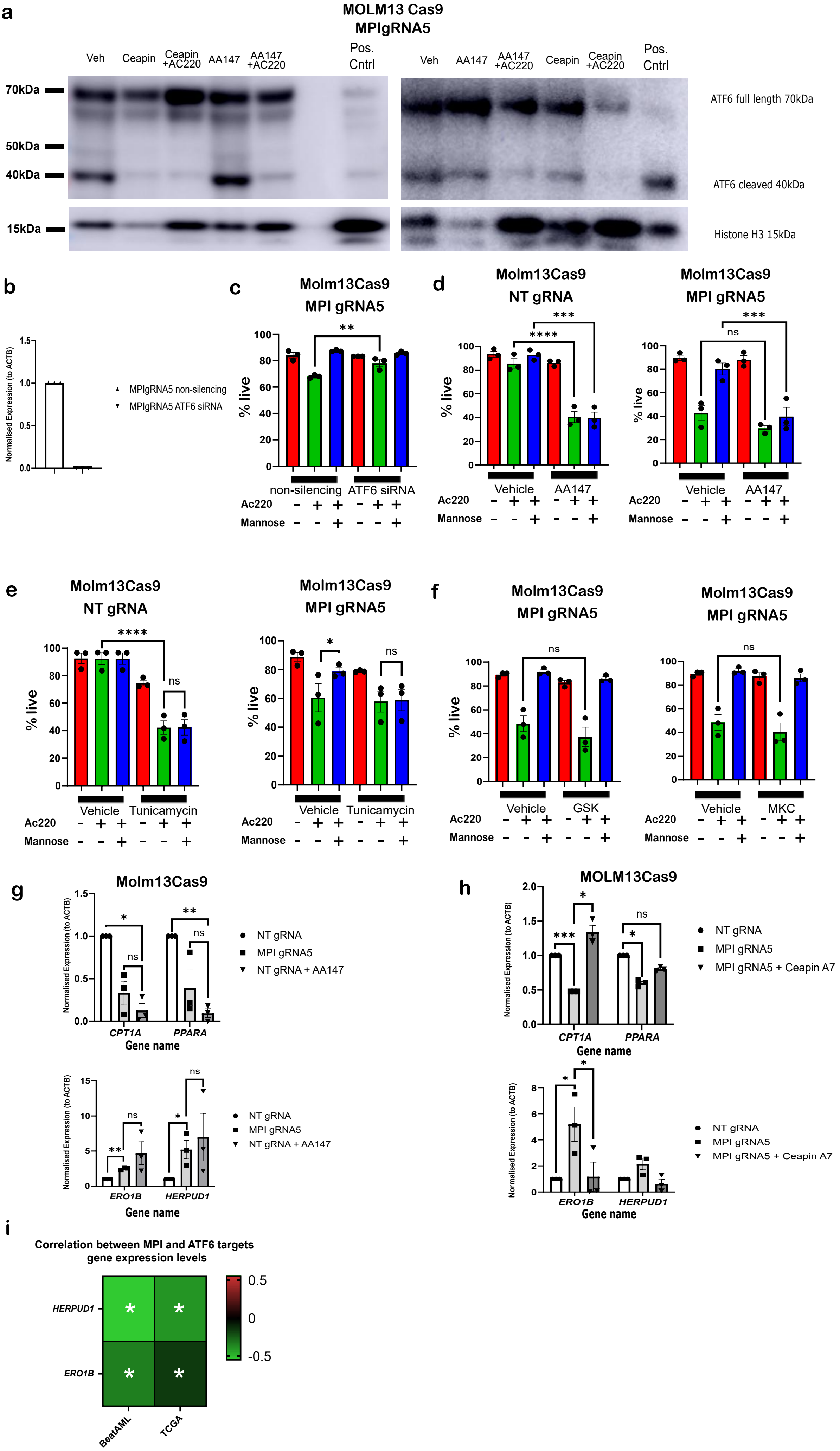
a
MOLM13 Cas9
MPIgRNA5
Veh
Ceapin
Ceapin +AC220
AA147
AA147 +AC220
Pos. Cntrl
Veh
AA147
AA147 +AC220
Ceapin
Ceapin +AC220
Pos. Cntrl
70kDa
50kDa
40kDa
15kDa
ATF6 full length 70kDa
ATF6 cleaved 40kDa
Histone H3 15kDa
b
Normalised Expression (to ACTB)
MPIgRNA5 non-silencing
MPIgRNA5 ATF6 siRNA
c
Molm13Cas9
MPI gRNA5
% live
non-silencing
ATF6 siRNA
Ac220
Mannose
d
Molm13Cas9
NT gRNA
Molm13Cas9
MPI gRNA5
% live
Vehicle
AA147
Ac220
Mannose
e
Molm13Cas9
NT gRNA
Molm13Cas9
MPI gRNA5
% live
Vehicle
Tunicamycin
Ac220
Mannose
f
Molm13Cas9
MPI gRNA5
Molm13Cas9
MPI gRNA5
% live
Vehicle
GSK
Vehicle
MKC
Ac220
Mannose
g
Molm13Cas9
Normalised Expression (to ACTB)
CPT1A
PPARA
Gene name
NT gRNA
MPI gRNA5
NT gRNA + AA147
ERO1B
HERPUD1
Gene name
h
MOLM13Cas9
Normalised Expression (to ACTB)
CPT1A
PPARA
Gene name
NT gRNA
MPI gRNA5
MPI gRNA5 + Ceapin A7
ERO1B
HERPUD1
Gene name
i
Correlation between MPI and ATF6 targets
gene expression levels
HERPUD1
ERO1B
BeatAML
TCGA
0.5
0
-0.5

**Supplementary figure 6**
**Inhibition of the ATF6 arm of ER stress helps reverse the phenotype of MPI KO**

**a –** Western blot of protein samples from NTgRNA or MPIgRNA5 MOLM13 cells treated with vehicle, AC220, AA147, Ceapin A7 or in combinations as indicated at 72 hours of treatment. Primary antibodies ATF6 and Histone H3 as a loading control, N=2; **b –** Relative expression of *ATF6* to *ACTB* expression, from RT-qPCR, in MPIgRNA5 MOLM13 cells transduced with 10nM ATF6 or non-silencing control siRNA, N=3; **c** - Percentage of live cells of MPIgRNA5 MOLM13 cells transfected with 10nM ATF6 or non-silencing siRNA for 48 hours then treated with vehicle, AC220 or AC220 and mannose as indicated for 72 hours. N=3, one-way Anova with Tukey's correction. **d** - Percentage of live cells of NTgRNA (left) or MPI gRNA5 (right) MOLM13 cells treated with vehicle, AC220, mannose or AA147 as indicated 72 hours after treatment. N=3, 1-way Anova with Tukey's correction; **e** - Percentage of live cells of NTgRNA (left) and MPIgRNA5 (right) MOLM13 cells treated with vehicle, AC220, mannose, tunicamycin or in combinations as indicated 72 hours after treatment. N=3, 1-way Anova with Tukey's correction; **f -** Percentage of live cells of MPIgRNA5 MOLM13 cells treated with vehicle, AC220, mannose, GSK2656157 (left panel) or MKC-3946 (right panel) as indicated after 72 hours. N=3, 1-way Anova with Tukey's correction; **g** - Relative expression of *CPT1A* and *PPARA* (top) and *ERO1B* and *HERPUD1* (bottom) normalised to *ACTB* expression, from RT-qPCR, in NTgRNA and MPIgRNA5 MOLM13 cells treated with vehicle or AA147 for 72 hours. N=3, 1-way Anova with Tukey's correction; **h** - Relative expression of *CPT1A* and *PPARA* (top) and *ERO1B* and *HERPUD1* (bottom) normalised to *ACTB* expression, from RT-qPCR, in NTgRNA and MPIgRNA5 MOLM13 cells treated with vehicle or CeapinA7 for 72 hours. N=3, 1-way Anova with Tukey's correction; **i** – Heatmap showing correlation between *MPI* and ATF6 target genes (*ERO1B* and *HERPUD1*) expression from the BeatAML (left column) and TCGA (right column) AML databases. Spearman's Rank correlation. For all panels, ns = not significant, *=p<0.05, **=p<0.01, ***=p<0.005, ****=p<0.001. All data presented as mean values +/- SEM.

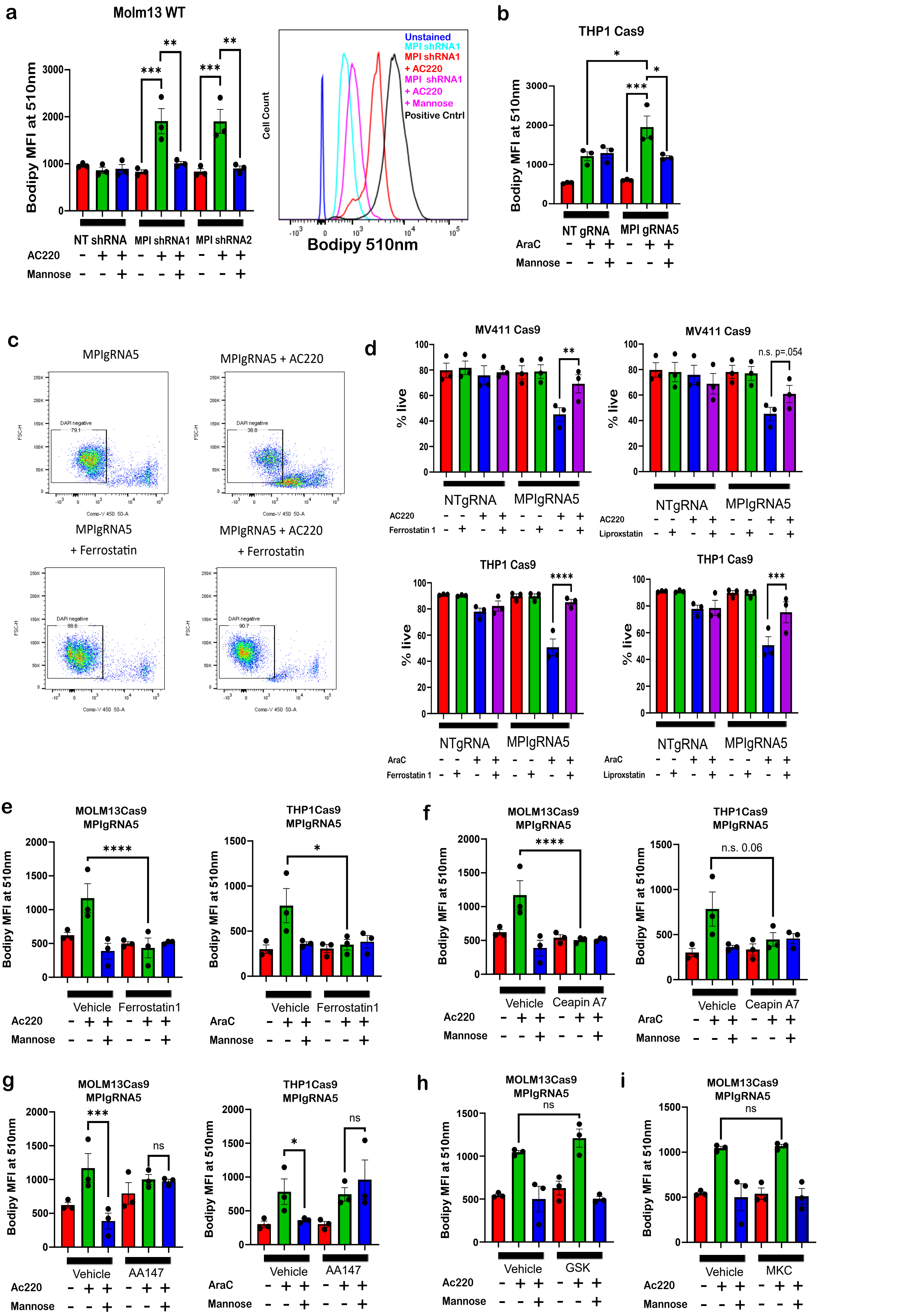

a
Molm13 WT
Bodipy MFI at 510nm
NT shRNA
MPI shRNA1
MPI shRNA2
AC220
Mannose
Unstained
MPI shRNA1
MPI shRNA1 + AC220
MPI shRNA1 + AC220 + Mannose
Positive Cntrl
Cell Count
Bodipy 510nm
b
THP1 Cas9
NT gRNA
MPI gRNA5
AraC
c
MPIgRNA5
MPIgRNA5 + AC220
MPIgRNA5 + Ferrostatin
MPIgRNA5 + AC220 + Ferrostatin
DAPI negative
79.1
38.8
88.6
90.7
FSC-H
Comp-V 450 50-A
d
MV411 Cas9
THP1 Cas9
% live
NTgRNA
MPIgRNA5
Ferrostatin 1
Liproxstatin
n.s. p=.054
e
MOLM13Cas9 MPIgRNA5
THP1Cas9 MPIgRNA5
Vehicle
Ferrostatin1
Ac220
f
Ceapin A7
n.s. 0.06
g
AA147
ns
h
GSK
i
MKC

**Supplementary figure 7**
**MPI KO AML cell lines are sensitive to ferroptotic cell death as a result of ATF6 activation**

**a** - MFI at 510nm of Bodipy 581/591-C11, which shows level of lipid peroxidation, in NTshRNA, MPIshRNA1 and MPI hRNA2 MOLM13 cells treated with vehicle, AC220 or mannose as indicated after 72 hours (left) with representative flow cytometry plot (right including positive control). N=3, 1 way Anova with Tukey's correction; **b** - Mean fluorescence intensity at 510nm of Bodipy 581/591-C11 in NTgRNA and MPIgRNA5 THP1 cells treated with AraC or mannose as indicated after 72 hours. N=3, 1-way Anova with Tukey's correction; **c** – Representative flow cytometry plots showing DAPI intensity vs FSC-H from MPIgRNA5 MOLM13 cells treated with AC220 and ferrostatin-1 as indicated for 6 days; **d -** Percentage of live NTgRNA and MPIgRNA5 MV411 or THP1 cells treated with vehicle, AC220, AraC, mannose, ferrostatin-1 (left), liproxstatin (right) or combinations as indicated for 6 days. N=3, 1-way Anova with Tukey's correction. **e -** MFI at 510nm of Bodipy 581/591-C11 in MPIgRNA5 MOLM13 and THP1 cells treated with AC220, AraC, mannose or ferrostatin1 as indicated after 72 hours. N=3, 1-way Anova with Tukey's correction; **f -** Mean fluorescence intensity at 510nm of Bodipy 581/591-C11, which shows level of lipid peroxidation, in MPI gRNA5 MOLM13 (left) and THP1 (right) cells treated with AC220, AraC, mannose or Ceapin A7 as indicated after 72 hours. N=3, 1-way Anova with Tukey's correction for multiple comparisons; **g -** Mean fluorescence intensity at 510nm of Bodipy 581/591-C11 in MPIgRNA5 MOLM13 (left) and THP1 (right) cells treated with AC220, AraC, mannose or AA147 as indicated after 72 hours. N=3, 1 way Anova with Tukey's correction for multiple comparisons; **h -** Mean fluorescence intensity at 510nm of Bodipy 581/591-C11 in MPI gRNA5 MOLM13 cells treated with AC220, mannose or GSK2656157 as indicated 72 hours after treatment. N=3, 1-way Anova with Tukey's correction; **i –** MFI at 510nm of Bodipy 581/591-C11 in MPIgRNA5 MOLM13 cells treated with AC220, mannose or MKC-3946 as indicated 72 hours after treatment. N=3, 1-way Anova with Tukey's correction. For all panels, ns = not significant, *=p<0.05, **=p<0.01, ***=p<0.005, ****=p<0.001, data presented as mean +/- SEM.

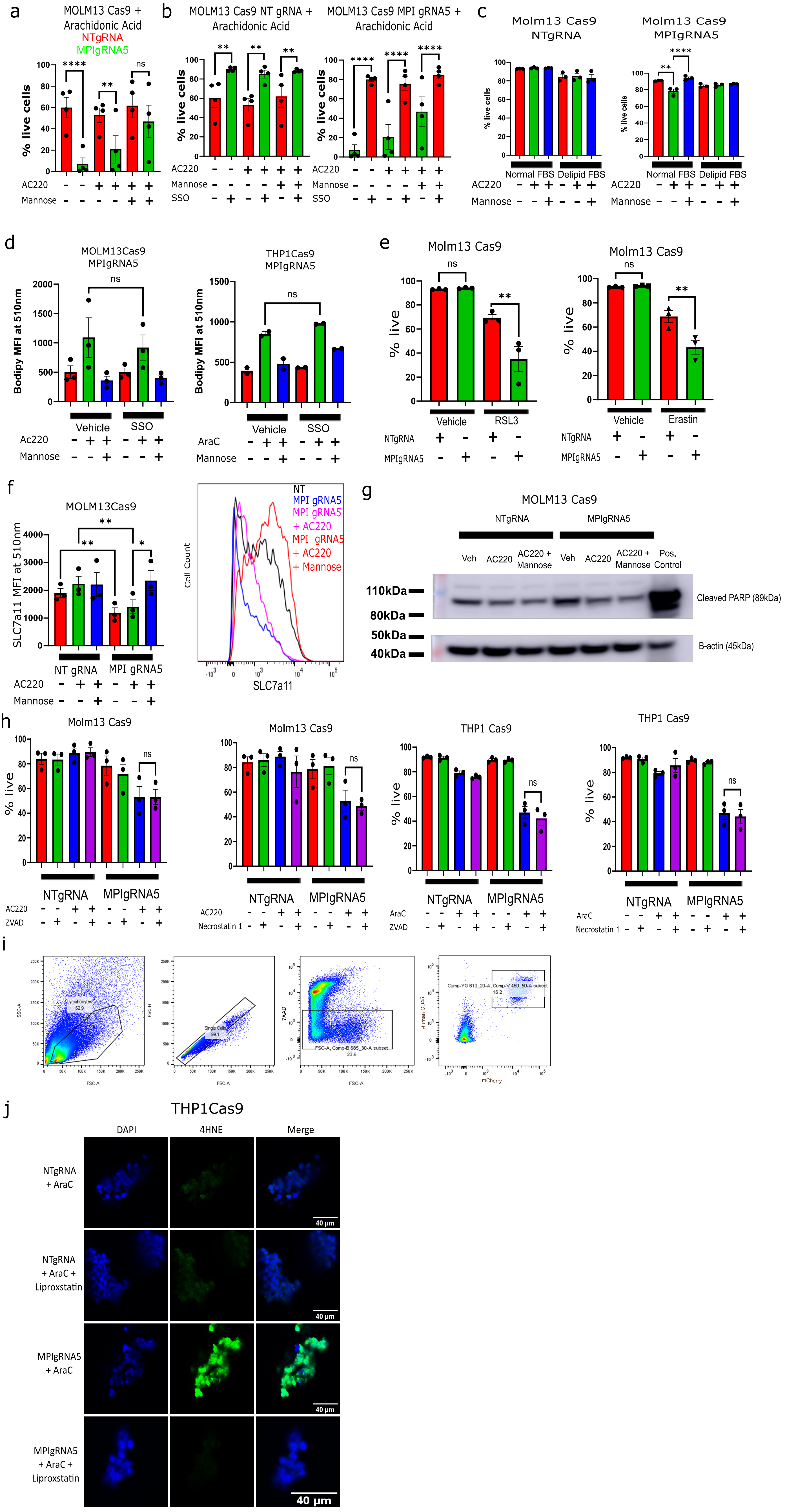
a
MOLM13 Cas9 +
Arachidonic Acid
NTgRNA
MPIgRNA5
% live cells
AC220
Mannose
b
MOLM13 Cas9 NT gRNA +
Arachidonic Acid
MOLM13 Cas9 MPI gRNA5 +
Arachidonic Acid
% live cells
AC220
Mannose
SSO
c
Molm13 Cas9
NTgRNA
Molm13 Cas9
MPIgRNA5
% live cells
Normal FBS
Delipid FBS
AC220
Mannose
d
MOLM13Cas9
MPIgRNA5
THP1Cas9
MPIgRNA5
Bodipy MFI at 510nm
Vehicle
SSO
Ac220
AraC
Mannose
e
Molm13 Cas9
% live
Vehicle
RSL3
Erastin
NTgRNA
MPIgRNA5
f
MOLM13Cas9
SLC7a11 MFI at 510nm
NT gRNA
MPI gRNA5
AC220
Mannose
NT
MPI gRNA5
MPI gRNA5
+ AC220
MPI gRNA5
+ AC220
+ Mannose
Cell Count
SLC7a11
g
MOLM13 Cas9
NTgRNA
MPIgRNA5
Veh
AC220
AC220 +
Mannose
Pos.
Control
110kDa
80kDa
50kDa
40kDa
Cleaved PARP (89kDa)
B-actin (45kDa)
h
Molm13 Cas9
THP1 Cas9
% live
NTgRNA
MPIgRNA5
AC220
AraC
ZVAD
Necrostatin 1
i
Lymphocytes
Single Cells
FSC-A
SSC-A
FSC-H
7AAD
Human CD45
mCherry
j
THP1Cas9
DAPI
4HNE
Merge
NTgRNA
+ AraC
NTgRNA
+ AraC +
Liproxstatin
MPIgRNA5
+ AraC
MPIgRNA5
+ AraC +
Liproxstatin
40 µm

**Supplementary figure 8**
**MPI KO AML cell lines are primed for ferroptosis via sensitivity to PUFAs**

**a** - Percentage of NTgRNA and MPIgRNA5 MOLM13 live cells treated with arachidonic acid in combination with vehicle, AC220 or mannose as indicated for 48 hours. N=4, ns = not significant, 1-way Anova with Tukey's correction; **b** - Percentage of NTgRNA and MPI gRNA5 MOLM13 live cells when treated with SSO or vehicle for 24 hours then arachidonic acid with vehicle, AC220 or mannose as indicated for a further 48 hours; N=4, 1-way Anova with Tukey's correction; **c** - Percentage of live NTgRNA or MPIgRNA5 MOLM13 cells cultured in either normal or delipidated FBS and treated with vehicle, AC220 or mannose as indicated 72 hours after treatment. N=3, 1-way Anova with Tukey's correction; **d** - MFI at 510nm of Bodipy 581/591-C11 in MPIgRNA5 MOLM13 and THP1 cells treated with AC220, AraC, mannose or SSO as indicated 72 hours after treatment. N=3, 1-way Anova with Tukey's correction; **e –** Percentage of live NTgRNA and MPIgRNA5 Molm13 cells treated with RSL3 or erastin after 48 hours. N=3, 1-way Anova with Tukey's correction; **f -** MFI from flow cytometry analysis of surface SLC7a11 on MOLM13 NTgRNA and MPIgRNA5 cells treated with vehicle, AC220 or mannose as indicated after 24 hours of treatment, N=3 1-way Anova with Tukey's correction. Representative plots of flow cytometry fluorescence intensity of MOLM13 NTgRNA and MPIgRNA5 cells with vehicle, AC220 or mannose as indicated (right); **g -** Western blot of cleaved PARP in MOLM13 NTgRNA and MPIgRNA5 treated with AC220 and mannose as indicated for 6 days. **h -** Percentage of NTgRNA and MPIgRNA5 MOLM13 or THP1 live cells treated with vehicle, AC220, mannose, ZVAD or necrostatin as indicated for 72 hours. N=3, 1-way Anova with Tukey's correction; **i -** Representative flow cytometry plots of the sorting of THP1 cells from transplanted NBSGW mice; **j** – Representative immunofluorescence images from NTgRNA or MPIgRNA5 THP1 cells sorted from transplanted NBSGW mice treated with AraC or liproxstatin as indicated. 2 fields of view taken for each independent animal, N=5 for MPIgRNA5 + AraC, N=4 for others. For all panels, ns = not significant, *=p<0.05, **=p<0.01, ***=p<0.005, ****=p<0.001, data presented as mean +/- SEM.

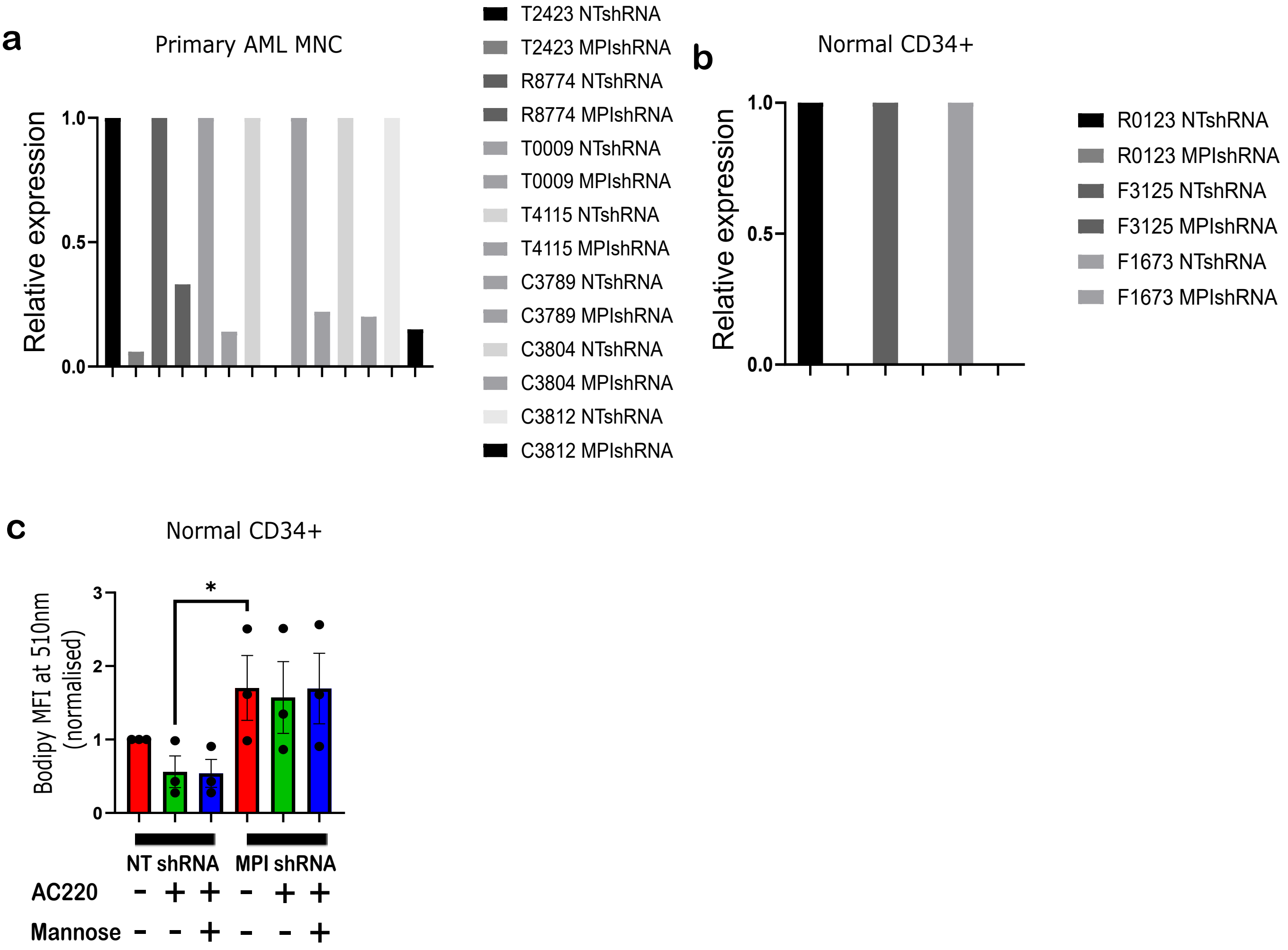
a
Primary AML MNC
Relative expression
1.0
0.5
0.0
T2423 NTshRNA
T2423 MPIshRNA
R8774 NTshRNA
R8774 MPIshRNA
T0009 NTshRNA
T0009 MPIshRNA
T4115 NTshRNA
T4115 MPIshRNA
C3789 NTshRNA
C3789 MPIshRNA
C3804 NTshRNA
C3804 MPIshRNA
C3812 NTshRNA
C3812 MPIshRNA
b
Normal CD34+
Relative expression
1.0
0.5
0.0
R0123 NTshRNA
R0123 MPIshRNA
F3125 NTshRNA
F3125 MPIshRNA
F1673 NTshRNA
F1673 MPIshRNA
c
Normal CD34+
Bodipy MFI at 510nm (normalised)
3
2
1
0
*
NT shRNA
MPI shRNA
AC220 - + + - + +
Mannose - - + - - +

**Supplementary figure 9**
**MPI knockdown efficiency in primary AML and CD34 samples**

**a** – Relative *MPI* expression measured by RT-qPCR in primary AML $FLT3^{ITD}$ samples transduced with either NT shRNA or MPI shRNA1; **b** - Relative *MPI* expression measured by RT-qPCR in normal CD34+ samples transduced with either NT shRNA or MPI shRNA1; **c** - Mean fluorescence intensity at 510nm of Bodipy 581/591 C11, which shows level of lipid peroxidation, in NT shRNA and MPI shRNA1 normal CD34+ cells treated with vehicle, AC220 (2.5nM) and mannose (100µM) in the indicated combinations at 72 hours after treatment. N=3 for each sample, 1 way Anova with Tukey's correction for multiple comparisons. For all panels, ns = not significant, *=p<0.05, **=p<0.01, ***=p<0.005, ****=p<0.001. All data presented as mean values +/- SEM.

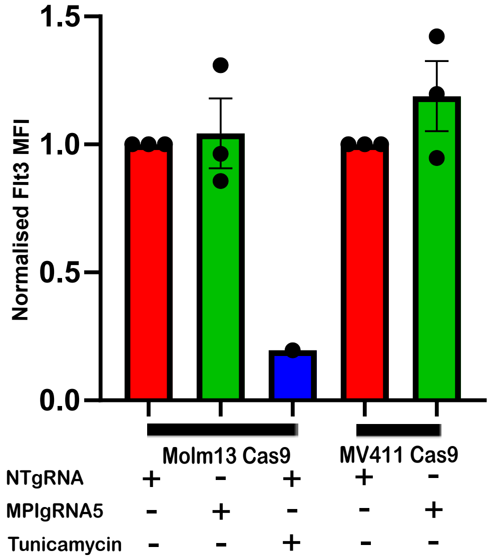

Normalised Flt3 MFI
1.5
1.0
0.5
0.0
Molm13 Cas9
MV411 Cas9
NTgRNA + - + + -
MPIgRNA5 - + - - +
Tunicamycin - - + - -

**Supplementary figure S10**
**MPI KO does not affect Flt3 surface expression**

MFI of surface Flt3 from flow cytometry of MPIgRNA5 or NTgRNA Molm13 or MV411 cells. Tunicamycin included in Molm13 cells as glycosylation inhibitor control. N=3 except tunicamycin treated where N=1.

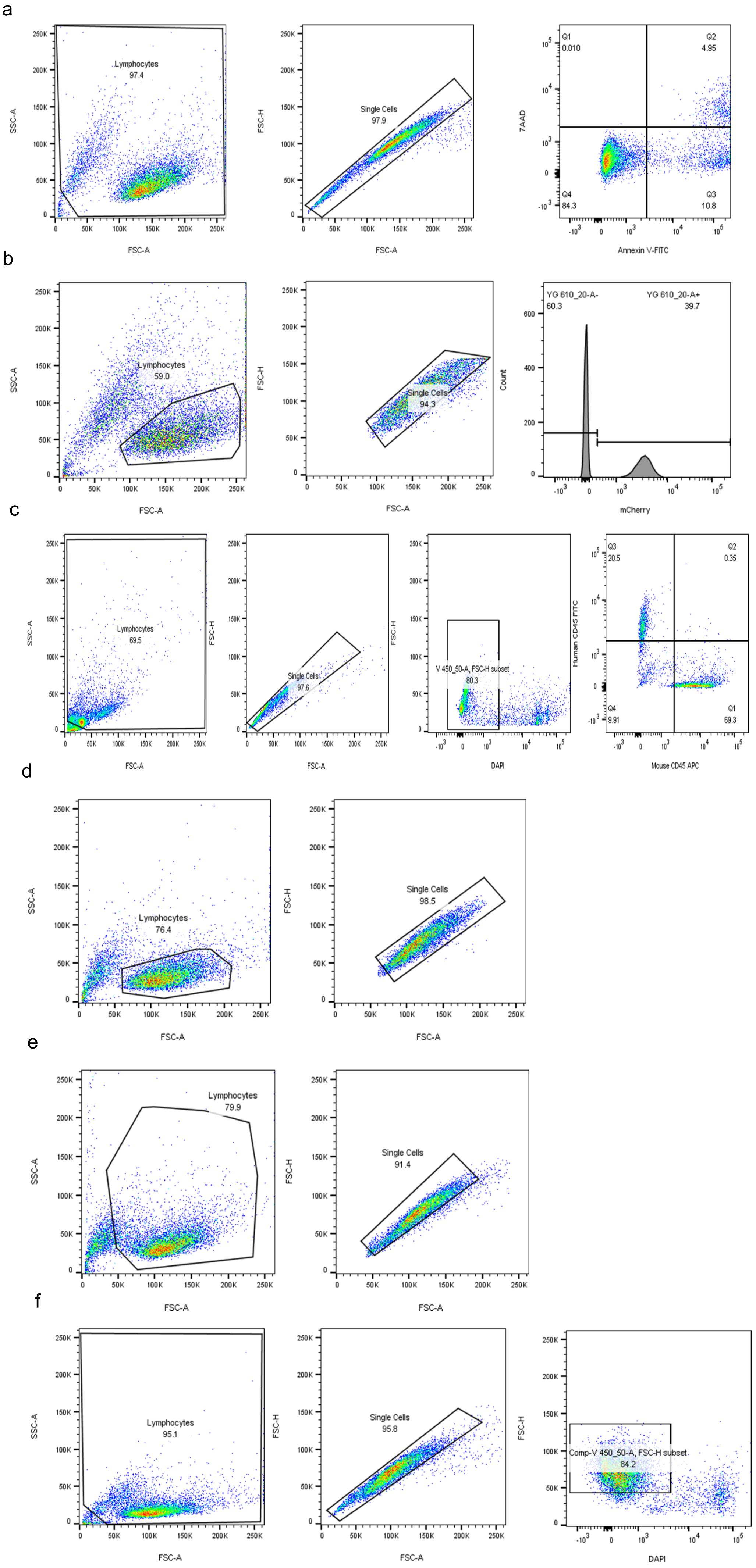

a
Lymphocytes
97.4
SSC-A
FSC-A
Single Cells
97.9
FSC-H
FSC-A
Q1
0.010
Q2
4.95
Q4
84.3
Q3
10.8
7AAD
Annexin V-FITC
b
Lymphocytes
59.0
SSC-A
FSC-A
Single Cells
94.3
FSC-H
FSC-A
YG 610_20-A-
60.3
YG 610_20-A+
39.7
Count
mCherry
c
Lymphocytes
69.5
SSC-A
FSC-A
Single Cells
97.6
FSC-H
FSC-A
V 450_50-A, FSC-H subset
80.3
FSC-H
DAPI
Q3
20.5
Q2
0.35
Q4
9.91
Q1
69.3
Human CD45 FITC
Mouse CD45 APC
d
Lymphocytes
76.4
SSC-A
FSC-A
Single Cells
98.5
FSC-H
FSC-A
e
Lymphocytes
79.9
SSC-A
FSC-A
Single Cells
91.4
FSC-H
FSC-A
f
Lymphocytes
95.1
SSC-A
FSC-A
Single Cells
95.8
FSC-H
FSC-A
Comp-V 450_50-A, FSC-H subset
84.2
FSC-H
DAPI

**FACS gating strategies**

**a** – Gating strategy for annexinV assays, used in figs 2c-e, 4f, 5e, 7a-b, supplementary figs 2f-h, 4h-I, 6d-f, 8a-c and h; **b** – Gating strategy for competition assays, used in figs 2a-b; **c** – Gating strategy for human/mouse CD45 assays, used in supplementary figs 2i-j; **d** – Gating strategy for all MFI-based assays (apart from Bodipy neutral lipid) used in figs 3c, 5b, 6c, 7c, supplementary figs 3d, g, 5a-b, 7a-b, e-I, 8d, f, 9c; **e** – Gating strategy for Bodipy neutral lipid stains used in figs 3d, supplementary figs 3h; **f** – Gating strategy for all DAPI based live cell assays used in figs 6d, 7e, supplementary figs 7d, 8e.

**Supplementary data 1 (in separate file)**

Global metabolomics of MPIgRNA5 and NTgRNA Molm13 cells treated with vehicle, AC220 or AC220 and mannose after 48 hours, N=4.

**Supplementary data 2 (in separate file)**

RNA-sequencing MPIgRNA5 and NTgRNA Molm13 cells treated with vehicle, AC220 or AC220 and mannose after 24 hours, N=2.

| **AML PATIENT SAMPLES** | | | | | | | | | |
|---|---|---|---|---|---|---|---|---|---|
| **PAT-ID** | **VIAL-ID** | **Sample Type** | **Diagnosis** | **Blast %** | **Sample Status** | **FLT3 (AR)** | **NPM1** | **Cytogenetics** | **Other mutations** |
| 7667 | R8774 | Peripheral Blood | AML | 74 | Diagnosis | ITD (low) | WT | 46, XX | None |
| 7667 | T0009 | Peripheral Blood | AML | 74 | Relapse | NA | NA | NA | NA |
| 8458 | T2423 | Bone Marrow | AML | 96 | Diagnosis | ITD | Mutant | 46, XX | DNMT3A, NRAS, IDH1 |
| 8458 | T4115 | Bone Marrow | AML | 96 | Relapse | NA | NA | NA | NA |
| 11630 | C3639 | Peripheral Blood | AML | 80 | Diagnosis | ITD (low) | Mutant | 46, XX | None |
| 11631 | C3638 | Peripheral Blood | AML | 90 | Diagnosis | ITD (high) | Mutant | 46, XY | IDH2 |
| 11658 | C3697 | Peripheral Blood | AML | 85 | Diagnosis | ITD (high) | WT | 46, XX | RUNX1, DNMT3A, TET2 |
| 11683 | C3754 | Peripheral Blood | AML | 90 | Diagnosis | ITD (high) | Mutant | 46, XX | DNMT3A |
| 11694 | C3789 | Peripheral Blood | AML | 86 | Diagnosis | ITD (high) | WT | 46, XX | CEBPA |
| 11699 | C3804 | Peripheral Blood | AML | 91 | Relapse | ITD (high) | Mutant | 46, XX, del9q | None |
| 11700 | C3812 | Peripheral Blood | AML | 90 | Diagnosis | ITD (low) | WT | 46, XX, del7q | DNMT3A, STAG2, IDH2, RUNX1 |

**NA, Not available**
**AR, Allelic Ratio**

| **NORMAL CD34$^{+}$ SAMPLES** | | | | | | | | | |
|---|---|---|---|---|---|---|---|---|---|
| **PAT-ID** | **VIAL-ID** | **Sample Type** | **Diagnosis** | **Blast %** | **Sample Status** | **FLT3 (AR)** | **NPM1** | **Cytogenetics** | **Other mutations** |
| 5972 | R0023 | Peripheral Blood CD34 | Myeloma | NA | Remission pre-autograft | NA | NA | NA | NA |
| 5255 | F3129 | Peripheral Blood CD34 | Myeloma | NA | Remission pre-autograft | NA | NA | NA | NA |
| 5228 | F1673 | Peripheral Blood CD34 | Myeloma | NA | Remission pre-autograft | NA | NA | NA | NA |

**NA, Not applicable**

**Supplementary table 1**

Information on primary patient samples used, including AML and normal CD34+ samples.

## S2a

Order (R-L) – Ladder, THP1 NT, THP1 MPIgRNA2, THP1 MPIgRNA5, Ladder, Molm13 NT, Molm13 MPIgRNA2, Molm13 MPIgRNA5, Ladder, MV411 NT, MV411 MPIgRNA2, MV411 MPIgRNA5

MPI

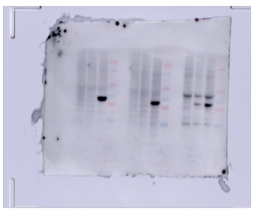

Histone H3

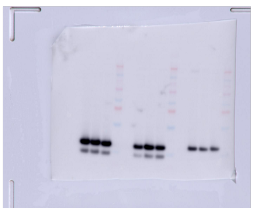

## S2e

Left blot unused, order (L-R) of right blot, Molm13: Ladder, NTshRNA, MPIshRNA1 (uninduced), MPIshRNA2 (uninduced), MPIshRNA1 (induced), MPIshRNA2 (induced),

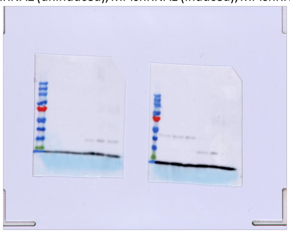

order (R-L) of right blot, THP1: Ladder, NTshRNA, MPIshRNA1 (uninduced), MPIshRNA2 (uninduced), MPIshRNA1 (induced), MPIshRNA2 (induced),

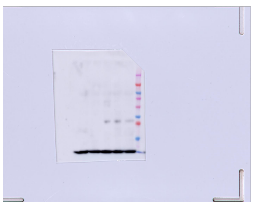

## S5f

Order (both, R-L) – Ladder, NT, NT Ac220, NT Ac220 mannose, MPI, MPI Ac220, MPI Ac220 mannose, positive

Total EIF2-alpha

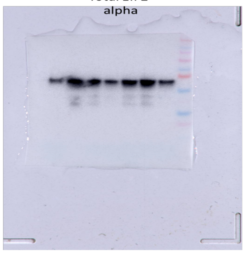

Phospho EIF2-alpha

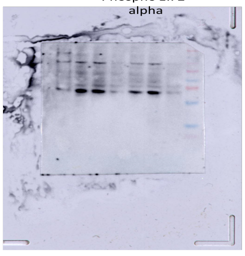

Order (both, L-R) – Ladder, NT, NT Ac220, NT Ac220 mannose, MPI, MPI Ac220, MPI Ac220 mannose, positive

Total EIF2-alpha

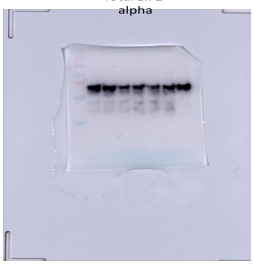

Phospho EIF2-alpha

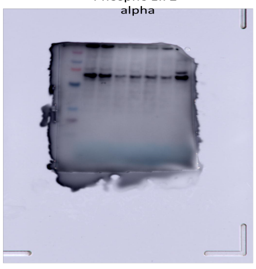

Order (both, R-L) – Ladder, NT, NT Ac220, NT Ac220 mannose, MPI, MPI Ac220, MPI Ac220 mannose, positive

Total EIF2-alpha

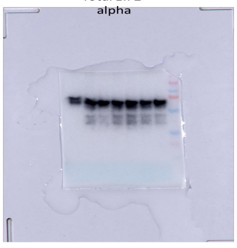

Phospho EIF2-alpha

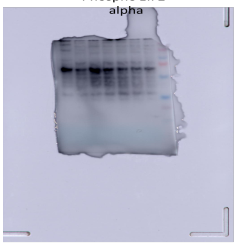

Order (total L-R) – NT, NT Ac220, NT Ac220 mannose, MPI, MPI Ac220, MPI Ac220 mannose, positive, ladder
Order (phospho L-R) – Ladder, positive, NT, NT Ac220, NT Ac220 mannose, MPI, MPI Ac220, MPI Ac220 mannose
Used in s5f

Total EIF2-alpha

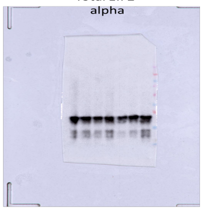

Phospho EIF2-alpha

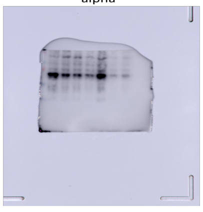

## S5e and S6a

Order (both, L-R) – Ladder, NT, NT Ac220, NT Ac220 mannose, MPI, MPI Ac220, MPI Ac220 mannose, Ladder, MPI, MPI Ceapin A7, MPI Ac220 Ceapin A7, MPI AA147, MPI Ac220 AA147, (empty well), positive control
Used in s5e

ATF6

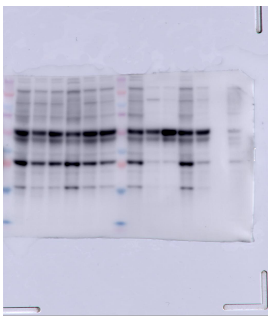

Histone H3

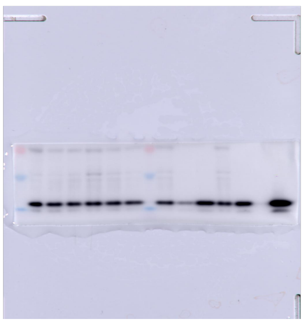

Order (both, R-L) – Ladder, NT, NT Ac220, NT Ac220 mannose, MPI, MPI Ac220, MPI Ac220 mannose, Ladder, NT, NT Ceapin A7, NT Ac220 Ceapin A7, NT AA147, NT Ac220 AA147, (empty well), positive control

ATF6

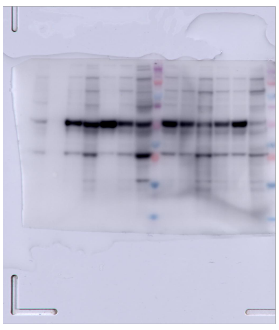

Histone H3

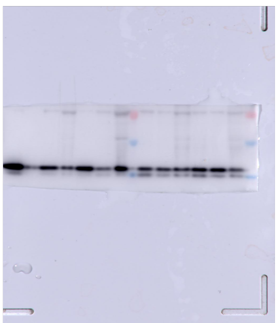

Order (L-R) – NT, NT Ac220, NT Ac220 mannose, MPI, MPI Ac220, MPI Ac220 mannose, positive, Ladder

ATF6

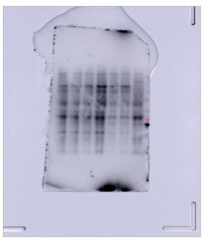

Order (R-L) – Ladder, NT, NT Ac220, NT Ac220 mannose, MPI, MPI Ac220, MPI Ac220 mannose, positive

ATF6

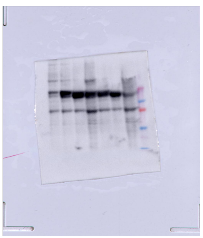

Order (L-R) – Ladder, NT, NT Ceapin A7, NT Ac220 Ceapin A7, NT AA147, NT Ac220 AA147,MPI, MPI Ceapin A7, MPI Ac220 Ceapin A7, MPI AA147, MPI Ac220 AA147, positive control
used in s6a

ATF6

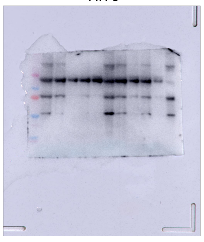

Histone H3

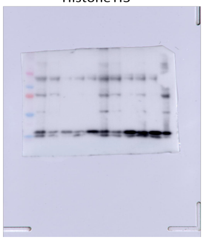

## S7g

Order (L-R) – Ladder, NT, NT Ac220, NT Ac220 mannose, MPI, MPI Ac220, MPI Ac220 mannose, positive, NT, NT Ac220, NT Ac220 mannose, MPI, MPI Ac220, MPI Ac220 mannose

PARP

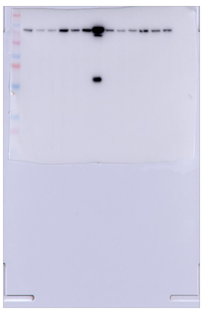

Actin

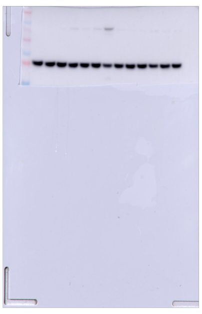

Order (R-L) – Ladder, NT, NT Ac220, NT Ac220 mannose, MPI, MPI Ac220, MPI Ac220 mannose, positive
Used in s7g

PARP

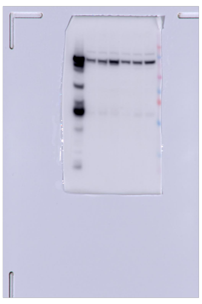

Actin

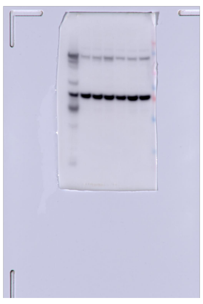

**Full Western Blot membranes**

Full membranes of western blots used either as panels in figures (as indicated) or for quantification purposes in this work.
