# Supplementary material for: Mannose metabolism inhibition sensitizes acute myeloid leukaemia cells to therapy by driving ferroptotic cell death
Source: Nat Commun. 2023 Apr 14;14:2132. doi: 10.1038/s41467-023-37652-0 (PMC10104861; doi:10.1038/s41467-023-37652-0)
Supplement: Supplementary file 3 — Description of Additional Supplementary Files [file 41467_2023_37652_MOESM3_ESM.pdf]

Description of Additional Supplementary Files

Supplementary Data 1 Description: Global metabolomics of MPIgRNA5 and NTgRNA Molm13 cells treated with vehicle, AC220 or AC220 and mannose after 48 hours, N=4.

Supplementary data 2 Description: RNA sequencing of MPIgRNA5 and NTgRNA Molm13 cells treated with vehicle, AC220 or AC220 and mannose after 24 hours, N=2.
