# Supplementary material for: Mannose metabolism inhibition sensitizes acute myeloid leukaemia cells to therapy by driving ferroptotic cell death
Source: Nat Commun. 2023 Apr 14;14:2132. doi: 10.1038/s41467-023-37652-0 (PMC10104861; doi:10.1038/s41467-023-37652-0)
Supplement: Supplementary file 6 — Reporting Summary [file 41467_2023_37652_MOESM6_ESM.pdf]

nature portfolio

Corresponding author(s): Paolo Gallipoli
Last updated by author(s): Mar 7, 2023

Reporting Summary

Nature Portfolio wishes to improve the reproducibility of the work that we publish. This form provides structure for consistency and transparency in reporting. For further information on Nature Portfolio policies, see our Editorial Policies and the Editorial Policy Checklist.

Statistics

For all statistical analyses, confirm that the following items are present in the figure legend, table legend, main text, or Methods section.

n/a	Confirmed	
☐	☒	The exact sample size (*n*) for each experimental group/condition, given as a discrete number and unit of measurement
☐	☒	A statement on whether measurements were taken from distinct samples or whether the same sample was measured repeatedly
☐	☒	The statistical test(s) used AND whether they are one- or two-sided<br>*Only common tests should be described solely by name; describe more complex techniques in the Methods section.*
☐	☒	A description of all covariates tested
☐	☒	A description of any assumptions or corrections, such as tests of normality and adjustment for multiple comparisons
☐	☒	A full description of the statistical parameters including central tendency (e.g. means) or other basic estimates (e.g. regression coefficient) AND variation (e.g. standard deviation) or associated estimates of uncertainty (e.g. confidence intervals)
☐	☒	For null hypothesis testing, the test statistic (e.g. *F*, *t*, *r*) with confidence intervals, effect sizes, degrees of freedom and *P* value noted<br>*Give P values as exact values whenever suitable.*
☒	☐	For Bayesian analysis, information on the choice of priors and Markov chain Monte Carlo settings
☒	☐	For hierarchical and complex designs, identification of the appropriate level for tests and full reporting of outcomes
☐	☒	Estimates of effect sizes (e.g. Cohen's *d*, Pearson's *r*), indicating how they were calculated

Our web collection on statistics for biologists contains articles on many of the points above.

Software and code

Policy information about availability of computer code

Data collection	BDS FACS Diva v9.0<br>Zeiss ZEN v3.0<br>Thermo Xcalibur 4.3.73.11<br>Agilent SEAHORSE Wave<br>Amersham 600 RGB imager (chemidoc)
Data analysis	FlowJo v10.6.1<br>GraphPad Prism 8<br>Microsoft Excel 2019<br>ImageJ 1.54<br>Thermo Tracefinder 4.1<br>Autoplotter

For manuscripts utilizing custom algorithms or software that are central to the research but not yet described in published literature, software must be made available to editors and reviewers. We strongly encourage code deposition in a community repository (e.g. GitHub). See the Nature Portfolio guidelines for submitting code & software for further information.

Data

Policy information about availability of data

All manuscripts must include a data availability statement. This statement should provide the following information, where applicable:

- Accession codes, unique identifiers, or web links for publicly available datasets
- A description of any restrictions on data availability
- For clinical datasets or third party data, please ensure that the statement adheres to our policy

Access to RNA-sequencing data is available at https://www.ebi.ac.uk/arrayexpress/experiments/E-MTAB-11750 (now publicly available)
Public datasets used are GSE6891 (https://www.ncbi.nlm.nih.gov/geo/query/acc.cgi?acc=GSE6891), E-TABM 1029 (https://www.ebi.ac.uk/biostudies/arrayexpress/studies/E-TABM-1029), GSE12417 (https://www.ncbi.nlm.nih.gov/geo/query/acc.cgi?acc=GSE12417), GSE15434 (https://www.ncbi.nlm.nih.gov/geo/query/acc.cgi?acc=GSE15434), GSE13159 (https://www.ncbi.nlm.nih.gov/geo/query/acc.cgi?acc=GSE13159), GSE10358 (https://www.ncbi.nlm.nih.gov/geo/query/acc.cgi?acc=GSE10358), GSE37642 (https://www.ncbi.nlm.nih.gov/geo/query/acc.cgi?acc=GSE37642), GSE76009 (https://www.ncbi.nlm.nih.gov/geo/query/acc.cgi?acc=GSE76009), GSE30377 (https://www.ncbi.nlm.nih.gov/geo/query/acc.cgi?acc=GSE30377), GSE83533 (https://www.ncbi.nlm.nih.gov/geo/query/acc.cgi?acc=GSE83533) and RNA-Seq from AML TCGA (data obtained from https://www.cbioportal.org/), BeatAML dataset (data obtained http://www.vizome.org/) and from manuscript 10.1056/NEJMoa1808777 (NEJM WUSM)

The following Data Availability Statement has been added to the manuscript

Data availability
The RNA-sequencing data generated in this study have been deposited in the ArrayExpress database and are available at https://www.ebi.ac.uk/arrayexpress/experiments/E-MTAB-11750
Untargeted metabolomics analysis is available in Supplemental Data 1
Source data are provided with this paper
All other data are available upon request to the corresponding author

Human research participants

Policy information about studies involving human research participants and Sex and Gender in Research.

Reporting on sex and gender	Sex and gender was not considered in study design due to the relatively small number of samples and presence of FLT3-ITD mutation in AML samples was the only variable that was deemed to be important for this work. Further, researchers had no access to information on sex or gender of patient samples due to anonymisation.
Population characteristics	As stated above, presence of FLT3-ITD mutation was present in all samples used. Other genotypic information is provided in Supplementary Table 1.
Recruitment	All newly diagnosed or relapsed patients are approached to donate samples to the BioBank and selection is based on patient consent.
Ethics oversight	East of England - Cambridge Research Ethics Committee REC 21/EE/0123 under title "Barts Haemato-Oncology Research Tissue Bank" IRAS project ID: 283103

Note that full information on the approval of the study protocol must also be provided in the manuscript.

Field-specific reporting

Please select the one below that is the best fit for your research. If you are not sure, read the appropriate sections before making your selection.

☒ Life sciences ☐ Behavioural & social sciences ☐ Ecological, evolutionary & environmental sciences

For a reference copy of the document with all sections, see nature.com/documents/nr-reporting-summary-flat.pdf

Life sciences study design

All studies must disclose on these points even when the disclosure is negative.

Sample size	No power calculation was performed for in vitro experiments. Number of independent repeats was determined based on previous experience of number of experiments required to reach a reliable conclusion and the inherent variability of cell lines. For in vivo experiment, number of mice per group were based on power calculations based on expected efficacy of treatment from in vitro data.
Data exclusions	No data was excluded.

Replication	The number of replicates mentioned in manuscript were performed, between 2 and 5 independent replicates for all experiments with exact numbers provided in figure legends. All experiments for the data presented, whether showing difference or not between the groups, were repeated independently to ensure reproducibility as per the sample size above.
Randomization	For in vivo work mice were randomised in treatment groups based on initial engraftment to ensure same level of disease at the start of therapy in each arm of the experiments. No randomisation was performed in other experiments.
Blinding	Researchers were not blinded as it was essential to know which treatments were required for each arm of experiments.

Reporting for specific materials, systems and methods

We require information from authors about some types of materials, experimental systems and methods used in many studies. Here, indicate whether each material, system or method listed is relevant to your study. If you are not sure if a list item applies to your research, read the appropriate section before selecting a response.

Materials & experimental systems

n/a	Involved in the study
☐	☒ Antibodies
☐	☒ Eukaryotic cell lines
☒	☐ Palaeontology and archaeology
☐	☒ Animals and other organisms
☒	☐ Clinical data
☒	☐ Dual use research of concern

Methods

n/a	Involved in the study
☒	☐ ChIP-seq
☐	☒ Flow cytometry
☒	☐ MRI-based neuroimaging

Antibodies

Antibodies used	annexin V-FITC (BioLegend, 640945) ATF6 (IF only, Santa Cruz biotech, SC166659) AlexFluor 488, goat anti-rabbit, (Thermo, A32731) anti-human CD45-FITC (BioLegend, 304005) anti-mouse CD45-APC (BioLegend, 157605), anti-SLC7a11 (SantaCruz Biotech, SC98552) Lectin FITC (Sigma, L0401) Histone H3 (Cell Signalling, 44995), MPI (Santa Cruz Biotech, SC393477), PARP (Cell Signalling, 56255), ATF6 (WB, Abcam, AB122897), EIF2-alpha (Cell Signalling, 9722), Phospho-EIF2-alpha (Cell Signalling, 9721), anti-4HNE antiserum (Alpha Diagnostic International, HNE11-S), anti-rabbit HRP (Cell Signalling, 7074), anti-mouse HRP (Cell Signalling, 7076)
Validation	annexin V-FITC - validated in cell lines by manifacturer as reactive to all mammalian species and authors using alternatives measurement of cell death (DAPI based cell death assays)<br>ATF6 (IF) - validated in various cell lines by manifacturer and by others in review section of antibody website and by authors using ATF6 inhibitor CeapinA7<br>AlexFluor 488, goat anti-rabbit - validated in various cell lines and primary antibodies for ICC by manifacturer<br>human CD45-FITC - validated for FC in cell lines by manifacturer and authors using murine and human cells<br>anti-mouse CD45-APC - validated for FC in cell lines by manifacturer and authors using human and murine cells<br>anti-SLC7a11 - validated in cell lines by manifacturer<br>Lectin FITC - Agglutination activity is expressed in µg/ml and is determined from serial dilutions in phosphate buffered saline, pH 7.3, of a 1 mg/ml solution. This activity is the lowest concentration to agglutinate a 2% suspension of human erythrocytes after 1 hour incubation at 25 °C as per manifacturer<br>total Histone H3 - validated on endogenous expression of Histone H3 by manifacturer<br>MPI - Validated on cell lines and primary tissue by manifacturer and authors in KO cells (see fig s2)<br>PARP - Validated by staurosporine treatment in multiple cell lines by manifacturer and by ourselves (see fig s8)<br>ATF6 (WB) - validated by ourselves with positive controls and using ATF6 inhibitor CeapinA7(see fig s5 and s6)<br>EIF2-alpha - validated by ourselves with positive controls (see fig s5)<br>Phospho-EIF2-alpha - validated by ourselves with positive controls (see fig S5)<br>anti-4HNE antiserum - validated by ourselves with positive controls (see Peer review reports)<br>anti-rabbit HRP - Specific species reactivity tested by manufacturer<br>anti-mouse HRP - Specific species reactivity tested by manufacturer

Eukaryotic cell lines

Policy information about cell lines and Sex and Gender in Research

Cell line source(s)	Molm13, MV411 and THP1 are established human cell lines obtained from Sanger Institute cancer cell collection. HEK293T-Phoenix cells were obtained from collaboration with the Huntly lab.<br>Primary sample information is provided in a supplemental table
Authentication	Cell lines were STR typed by Eurofins Genomics before start of project and again in 2021 for confirmation
Mycoplasma contamination	Cell lines tested negative for mycoplasma contamination at 3 month intervals
Commonly misidentified lines (See ICLAC register)	none

Animals and other research organisms

Policy information about studies involving animals; ARRIVE guidelines recommended for reporting animal research, and Sex and Gender in Research

Laboratory animals	NBSGW (NOD.Cg-KitW-41J Tyr + Prkdcscid Il2rgtm1Wjl/ThomJ) mice and NSG (NOD.Cg-PrkdcscidIl2rgtm1Wjl/SzJ) mice, aged 8-12 weeks old at point of transplant.
Wild animals	None
Reporting on sex	No sex based analyses performed
Field-collected samples	None
Ethics oversight	Individual experiments were approved by our local mouse establishment (BSU), Charterhouse Sq, QMUL. All experiments were performed under UK Home Office Personal Project Licence<br>Number: PP4153210 | Granted: 04 Feb 20 approved by the Home office of the United Kingdom.

Note that full information on the approval of the study protocol must also be provided in the manuscript.

Flow Cytometry

Plots

Confirm that:

☒ The axis labels state the marker and fluorochrome used (e.g. CD4-FITC).

☒ The axis scales are clearly visible. Include numbers along axes only for bottom left plot of group (a 'group' is an analysis of identical markers).

☒ All plots are contour plots with outliers or pseudocolor plots.

☒ A numerical value for number of cells or percentage (with statistics) is provided.

Methodology

Sample preparation	Cells harvested and washed 3x in pbs, samples stained as per methods according to the type of antibody used and experiments
Instrument	BDS biosciences Fortessa and Symphony instruments
Software	FlowJo v10.6.1
Cell population abundance	Example sorting strategy has been provided in the figures where relevant. Cell population % information was not analysed as it was not necessary due to the nature of the experiment (we only needed to isolate the cell populations of interest to perform downstream experiments).
Gating strategy	Gating strategy varied according to the experiments. For viability experiments single cells taken from FSC-A vs FSC-H and assigned live or dead based on single colour positive controls for live (unstained) and dead (stained cells). For all other experiments, live cells assigned based on FSC-A vs SSC-A, single cells taken from FSC-A vs FSC-H and gating was based on single stained positive versus negative controls for each individual fluorochrome in each experiment. For further information on all gating strategies used, see supplementary information.

☒ Tick this box to confirm that a figure exemplifying the gating strategy is provided in the Supplementary Information.
